# Prevalence, diagnosis and treatment of ADHD in Arab and Jewish children in Israel, where are the gaps?

**DOI:** 10.1186/s12888-023-05090-3

**Published:** 2023-08-11

**Authors:** Amal Shehadeh-Sheeny, Orna Baron-Epel

**Affiliations:** School of Public Health, Faculty of Social Welfare and Health Studies, University of Haifa, Mount Carmel, Haifa, 31905 Israel

**Keywords:** Attention-deficit / hyperactivity disorder, Diagnosis, Education, Ethnicity, Socio-economic status, Help-seeking

## Abstract

**Background:**

Attention-deficit/hyperactivity disorder (ADHD) is the most common neurobehavioral disorder affecting children and causing significant impairment. It is not clear to what extent ADHD differs between population groups. This study aims to assess prevalence, diagnosis and treatment of ADHD among Arab and Jewish children of primary school age in Israel.

**Methods:**

Cross-sectional survey, including 517 parents of children ages 7–10 (225 Jewish and 292 Arab) and 60 homeroom teachers of the corresponding children. Both parents and homeroom teachers completed the ADHD Rating Scale-V-RV. ADHD was defined according to DSM-5 ADHD criteria by both parents and teachers, or clinical diagnosis. In addition, parents reported ADHD medication and adherence to medication.

**Results:**

Prevalence of ADHD was similar for both groups. Yet, seeking diagnosis was lower among Arab Muslim children (9.2%) compared to Jewish children (17.8%). Arab Muslim children received significantly less medication compared to Jewish children. Parental decision to seek diagnosis was associated with education (OR = 6.14, CI 1.74–21.71), not ethnicity. Ethnicity predicted parents’ decisions to pharmacologically treat their children with ADHD (OR = 7.61, CI 1.14–50.86) and adherence to medication (OR = 10.19, CI 1.18–88.01).

**Conclusion:**

Education is critical in the help-seeking process, affecting the rate of ADHD diagnosis. Pharmacological treatment and adherence are correlated with ethnicity. Parents with limited education and minorities should be targeted for interventions to increase awareness regarding ADHD and treatment.

## Background

Attention-Deficit/Hyperactivity Disorder (ADHD) is the most common and most studied neurodevelopmental disorder of childhood [1]. ADHD is defined as a “persistent disorder of inattention and/or hyperactivity-impulsivity that interferes with functioning or development”, and is generally subdivided to inattentive, hyperactive or combined type [2, 3]. Children with ADHD are more likely to experience a variety of negative outcomes on academic performance, social relationships, emotional and behavioral difficulties, and occupational functioning [2].

The reported prevalence of ADHD in children ranges from 2 to 18% depending upon the diagnostic criteria and the population studied [1, 4]. Prevalence of ADHD in school-age children is estimated to be between 9% and 15% [4]. The prevalence of ADHD may be related to social and cultural characteristics [5–8]. There is a worldwide increase in rates of diagnosis and pharmacological treatment of ADHD in recent years [9]. The increase in incidence, prevalence and pharmacological treatment of ADHD may be attributed to changing attitudes towards the disorder and its treatment, improved awareness and case-identification by primary care professionals, homeroom teachers, and parents [10].

There is no definitive medical test or biological marker that confirms the presence of the disorder [5]. ADHD symptoms are not always apparent in new, unfamiliar and highly structured settings, which makes observations of the child during an office visit less valuable than histories obtained from the parent and teacher [5, 11]. ADHD is usually diagnosed following administration of a variety of behavioral questionnaires for the assessment of ADHD, as many of which are based on the criteria of the Diagnostic and Statistical Manual for Mental Disorders DSM-5 [2]. Three subtypes of ADHD listed in the DSM-5 can be diagnosed: predominantly inattentive, predominantly hyperactive and impulsive, and a combined category for those who are both inattentive and impulsive/hyperactive. Diagnosis of ADHD requires that both symptoms and impairment from those symptoms are “pervasive” and present in at least two settings, e.g. at school and at home. Thus, clinicians and researchers rely heavily on reports by parents and teachers regarding a child’s behavior when identifying ADHD. Teachers are particularly valuable informants because they usually have a good sense of developmentally appropriate behavior [12].

In general, treatment for children with ADHD has expanded considerably and there is significant progress toward standardization of assessment and diagnostic procedures [13]. Nevertheless, there is substantial underdiagnosis and treatment of ADHD among children in ethnic minorities [14]. Early identification, intervention and appropriate support can greatly improve the outcomes and quality of life for children with ADHD [15]. Thus, a comprehensive understanding of ADHD across diverse populations is crucial for accurate diagnosis, equitable access to care, culturally sensitive interventions and addressing health disparities [16].

In Israel, according to the Ministry of Health’s regulations, only a specialist in pediatric neurology and child development, a specialist in pediatric and youth psychiatry, a specialist in adult neurology or psychiatry, and a pediatrician with at least 3 years’ experience in child development, can diagnose a child with ADHD. This is performed usually according to the most recent DSM criteria. In fact, the essential components of the diagnostic procedure include meeting DSM criteria and evaluation following completion of a validated diagnostic questionnaire by parents and teachers [17].

Medical services, are available for all Israeli residents by law. In practice, however, socioeconomically disadvantaged population groups and peripheral populations suffer from poor access to these services [18].

Most studies on the prevalence of ADHD in school age children in Israel were conducted by examination of stimulant medication prescriptions which are recommended as a first line treatment [19–21]. The percentage of Arab children who were prescribed stimulant medications was considerably lower than Jewish children, who received four times more prescriptions for methylphenidate than Arab children [21]. However, the number of prescriptions given to children is not an accurate measure for the prevalence of ADHD since even children prescribed only a few tablets (i.e., for the performance of a diagnostic test) are registered as receiving pharmacological treatment for ADHD. Moreover, not all children with ADHD are prescribed methylphenidate and not all parents of children with ADHD reach out for professional diagnosis [22].

As observed in other studies, over one third of children with ADHD do not use stimulants and about one third of children prescribed stimulants are not diagnosed with ADHD [23]. The differences between Jewish and Arab children regarding the rate of prescriptions of stimulants suggest the need for studies that examine whether ADHD is indeed more common among Jewish children, or whether fewer Arab children turn to medical services for diagnosis and treatment.

The purpose of our study was to assess the prevalence of ADHD among elementary school age children in representative Arab and Jewish populations in Northern Israel, by using the DSM-5 criteria for ADHD. We also assessed the prevalence of help seeking for medical diagnosis, pharmacological treatment and adherence to medication. In addition, we studied the concordance between teachers’ and parental assessments in both populations.

## Methods

### Design and setting

The study was a cross-sectional telephone survey targeting Arab and Jewish parents in the Northern region of Israel. The research protocol was approved by the Ethics Committees of the Ministry of Education and the University of Haifa. The same sampling techniques were utilized in order to obtain representative samples from each population, drawing a random stratified cluster sample from each population. First, ten villages and towns were randomly selected in the Northern district based on ethnicity (Jewish and Arab) and socioeconomic level [24]. Second, based on sample size requirement and in order to ensure representation from high, medium and low socioeconomic schools among both Arab and Jewish populations, 26 schools located in these villages and towns were randomly sampled using lists of schools obtained from the Israel Ministry of Education, keeping a ratio of 1:1 between Arab and Jewish schools. It is important to note that the Israeli educational system is divided into Arab and Jewish schools. Consent was obtained from 15 Arab and Jewish school principals. After obtaining consent from the principals, parents were sent a letter explaining the study and a consent form for participation in the study. Homeroom teachers of the relevant classes were approached by the principals and their consents were obtained. From each school, we sampled four classes, one class from each grade (second to fifth grade). All parents in a participating class were sampled. The response rate for Arab parents was 64.5%, and 54% for the Jewish parents. In addition, all Arab and Jewish homeroom teachers of the relevant classes have participated.

### Participants

The study was conducted from October 2021 through May 2022. The survey included 517 parents of children aged 7–10 (from second through fifth grade), in 15 Arab and Jewish elementary schools in Northern Israel (292 Arab Muslims and 225 Jewish children). All parents and 60 homeroom teachers of these children (28 Jewish and 32 Arab homeroom teachers) provided written informed consent for participation in the study. Twenty-one questionnaires were not completed and were therefore excluded from analysis. All Arab participants in the study were Muslims.

### Research tools

The ADHD– Rating Scale-V (ADHD-RS-V) questionnaire includes 18 items based on the diagnostic criteria for ADHD as described in the fifth version of the Diagnostic and Statistical Manual of Mental Disorders published by the American Psychiatric Association in 2013 (DSM-5) [2]. Each item/ symptom was rated according to its severity in the child on a four-point Likert scale (0 = Never or rarely; 3 = Very often). Ratings 2 and 3 are considered positive, as is common in the literature [25]. The tool examines the components of the disorder, confirms its existence and classifies the attention deficit disorder into three types: type 1- inattention, 2-hyperactivity and impulsivity, 3-combined. The items/ symptoms are divided into inattention (9 symptoms), and hyperactivity and impulsivity (9 symptoms). In order to be classified as type 1 or type 2, at least six out of nine symptoms must have been present during the previous 6 months. If there are six or more symptoms for the two categories, the type of disorder is considered combined. The ADHD– Rating Scale-V (ADHD-RS-V) was translated to Hebrew and Arabic by experts in the above languages, back-translated to English to validate the quality of the translation. Parents from the Arab schools answered the questionnaire in Arabic and parents from the Jewish schools answered the questionnaire in Hebrew. In our sample, Coefficient Alpha for the Jewish parent scales (Hebrew language) and the Arab parent scales (Arabic language) was 0.91 and 0.87 respectively. And coefficient alpha for the Jewish teacher scales and Arab teacher scales were 0.95 and 0.94, respectively.

As mentioned before, both parents and homeroom teachers completed the ADHD– Rating Scale-V (ADHD-RS-V) questionnaire for each child. The interviews with the homeroom teachers were conducted to identify children at high risk for ADHD, in order to validate parental reports regarding the possible presence of ADHD in their children. We purposefully chose the second and third trimesters of the school year in order to make sure that the teachers were well acquainted with their pupils.

A positive ADHD diagnosis was considered according to the ADHD–Rating Scale-V (ADHD-RS-V) if both parent and teacher endorsed at least 6 of 9 inattention or hyperactivity/impulsivity symptoms with a score of 2 or higher (presence of impairment in two settings: home and school), or if parents reported that their child was clinically diagnosed by a physician, including a pediatric neurology and child development specialist, a specialist in pediatric and youth psychiatry, a specialist in adult neurology or psychiatry, and a pediatrician with at least 3 years’ experience in child development.

The survey for parents also included: Parents’ Socio-demographic variables: sex, age, town, education, income level, number of children, marital status, ethnicity, religion and employment status. Education was coded as non-academic education (1) and academic education (2). Parental income: Self-reported family income (parent report) was measured by three levels: below, equal to, or above the average net monthly income per family per household in Israel according to the data of the Central Bureau of Statistics. These were then grouped into two categories: Low to medium income levels and high- income level. Marital status was reported as married or living with spouse (1); and single, divorced or widowed (0). Employment was dichotomized as: employed (1) and unemployed (0). Participants were also asked to define their ethnicity as Jewish or Arab.

In addition, the parent’s questionnaire included questions concerning seeking diagnosis and medication. “Have you approached a specialist seeking ADHD diagnosis for your child?” “Has your child been diagnosed with ADHD?” “Did the doctor recommend medication for your child?” “Does your child receive medication for ADHD?” In addition, questions concerning the child’s adherence to medication (for children diagnosed with ADHD): the parents were asked to indicate whether they followed the doctor’s recommendations on a four-point scale (4 = very often, 1 = never) and whether the child opposed taking the medication on a scale ranging from 1-very often to 4-never.

### Statistical analysis

We used descriptive statistics to characterize the study sample. Chi square and Fisher’s tests were used to examine the variables associated with the parents’ decision to approach a doctor to seek clinical diagnosis for their children, the parents’ decision to treat with medication and adherence to medication. Finally, we ran multivariable logistic regression models, presenting odds ratios, confidence intervals and p-values. The variables added to the regression model were those associated with the dependent variables and that had low multicollinearity with other variables in the model. Analyses were performed using SPSS version 27.

## Results

The parents’ sample included 292 Arab Muslim parents and 225 Jewish parents. The average age of Arab Muslim parents was 38.0 years, and 43.6 for Jewish parents. There were significant between group differences regarding socio-economic characteristics (education levels, occupations and economic situations). More Jewish parents had academic education (95.6% compared to 43.8% of the Arab parents). Lower income levels were reported by Arab parents (38.4% of the Arab parents belonged to families with low-income levels, compared to 2.7% of the Jewish parents). Jewish parents had a lower average number of children (2.78 for Jewish families compared to 3.36 in the Arab families), and a higher percentage were employed (96% of the Jewish parents compared to 50.7% of the Arab parents) (Table 1).

**Table 1:** Parents’ characteristics by ethnicity, percent and number

Characteristics			Jewish n = 225	Arab (Muslims) n = 292	All Participants n = 517	P- value
**Sex**	Fathers		2.7% (6)	1% (3)	1.7% (9)	0.158
	Mothers		97.3% (219)	99% (289)	98.3% (508)	
**Average age (years)**	Parents		43.57	37.95	40.4	< 0.001
**Parents’ education**	Academic		95.6% (215)	43.8% (128)	66.3% (343)	< 0.001
	Non-Academic		4.4% (10)	56.2% (164)	33.7% (174)	
**Marital status**	Lived with a partner		94.7% (213)	97.9% (286)	96.5% (499)	0.044
	Lived without a partner		5.3% (12)	2.1% (6)	3.5% (18)	
**Average number of children**			2.78	3.36	3.11	< 0.001
**Employment**		Employed	96% (216)	50.7% (148)	70.4% (364)	< 0.001
		Unemployed	4% (9)	49.3% (144)	29.6% (153)	
**Income level**	Low		2.7% (6)	38.4% (112)	22.8% (118)	< 0.001
	Medium		5.8% (13)	33.6% (98)	21.5% (111)	
	High		91.6% (206)	28.1% (82)	55.7% (288)	

### Prevalence of ADHD

Of the 517 children reported on by their parents and homeroom teachers, 255 were boys (49.3%) and 262 were girls (50.7%) from second grade through fifth grade. Of the 517 children screened, 153 children (82 Arab children and 53 Jewish children) fulfilled at least one of the three diagnostic criteria based on the parent’s questionnaire, teacher’s questionnaire, or a doctor’s clinical diagnosis. Of these, 88 children (49 Arab, 39 Jewish) met the diagnostic criteria of DSM-5 based on both parents’ and teachers’ questionnaires. In addition, six children were clinically diagnosed with ADHD by a doctor, however with only one questionnaire suggestive of ADHD (completed by parents or teachers). A total of 94 children (18.2%) were categorized as having ADHD in this study sample (Table 2). Concordance between parents’ questionnaire and teachers’ questionnaire was 73.6% in the Jewish sample and 60% in the Arab Muslim sample, P < 0.001.

Table 2 presents the frequencies of children with ADHD by ethnic groups. There were no significant differences in prevalence of ADHD among the two ethnic groups, 18.7% for Jewish children and 17.8% for Arab children (P = 0.80). Yet, parents who approached a doctor seeking clinical diagnosis for their children was much lower among Arab children (9.2%) compared to 17.8% of the Jewish parents (P = 0.004). Thus, only 4.8% of the Arab children were clinically diagnosed compared to 13.8% of the Jewish children (P < 0.001). Moreover, only 1% of the Arab parents reported that their children were treated with medication, compared to 6.7% of the Jewish children (P < 0.001).

Among ADHD positive children (N = 94), 76.2% of the Jewish parents approached a doctor seeking clinical diagnosis for their children, compared to only 36.5% of the Arab children (P < 0.001). Thus, more Jewish children were diagnosed (73.8%), compared to 26.9% of the Arab children (P < 0.001), and more Jewish children received prescriptions for pharmacological treatment (42.9% versus 17.3% of the Arab children, P = 0.006). In addition, a higher percent of Jewish parents reported treating their children diagnosed with ADHD with medication (35.7% vs. 5.7% of the Arab parents, P < 0.001). Yet, 4.8% of the Jewish children with ADHD reported discontinuing the pharmacological treatment because of the medication adverse effects, and 1.9% of the Arab children discontinued treatment, because the child opposed taking the medication. Significantly, higher adherence with medication was reported by parents of Jewish children as 72.2% of the Jewish children, which were prescribed medication, adhered to the medication compared to 22.2% of the Arab children.

The prevalence of ADHD subtypes: 68.1% had predominantly Inattentive type, 9.6% had predominantly Hyperactive-Impulsive type and 22.3% combined type (Inattentive and Hyperactive-Impulsive type), with more Jewish children having the Inattentive type, and more Arab children having the Hyperactive-Impulsive and combined types (see Table 2).

**Table 2:** Frequencies of children with ADHD by ethnic groups

		All participants N = 517		Jewish N = 225		Arab Muslims N = 292		*P- value
		% (N)		% (N)		% (N)		
**Meeting criteria of ADHD as reported by (N = 517):**								
Parents		24.8 (128)		23.1 (52)		26 (76)		0.44
Homeroom teachers		25.9 (134)		24 (54)		27.4 (80)		0.38
Parents and homeroom teachers		17.3 (88)		17.3 (39)		16.8 (49)		0.86
Parents and homeroom teachers, and or clinically diagnosed		18.2 (94)		18.7 (42)		17.8 (52)		0.80
**Parents reporting that their child (N = 517)**:								
Approached the doctor seeking clinical diagnosis		13 (67)		17.8 (40)		9.2 (27)		0.004
Has a clinical diagnosis		8.7 (45)		13.8 (31)		4.8 (14)		< 0.001
Received prescription for medication		5.2 (27)		8 (18)		3.1 (9)		0.013
Received pharmacological treatment		3.4 (18)		6.7 (15)		1.02 (3)		< 0.001
Adhered to medication		2.9 (15)		5.8 (13)		0.7 (2)		< 0.001
**Frequencies among children meeting criteria for ADHD (ADHD positive children) (N = 94)**								
		All Participants N = 94		Jewish participants N = 42		Arab participants N = 52		
Sex	Boys	67 (63)		59.5 (25)		73.1 (38)		< 0.001
	Girls	33 (31)		40.5 (17)		26.9 (14)		
Grade	Second grade	16 (15)		14.3 (6)		17.3 (9)		0.52
	Third grade	18.1 (17)		14.3 (6)		21.2 (11)		
	Fourth grade	30.9 (29)		31 (13)		30.8 (16)		
	Fifth grade	35.1 (33)		40.5 (17)		30.8 (16)		
Inattentive type		68.1 (64)		76.2 (32)		61.5 (32)		< 0.001
Hyperactive-Impulsive type		9.6 (9)		4.8 (2)		13.5 (7)		0.003
Inattentive & Hyperactive-Impulsive type		22.3 (21)		19 (8)		25 (13)		< 0.001
**Parents reporting that their child (N = 94)**:								
Approached the doctor seeking clinical diagnosis		54.3 (51)		76.2 (32)		36.5 (19)		< 0.001
Clinically diagnosed		47.9 (45)		73.8 (31)		26.9 (14)		< 0.001
Received prescription for medication		28.7 (27)		42.9 (18)		17.3 (9)		0.006
Treated with medication		19.1 (18)		35.7 (15)		5.7 (3)		< 0.001
Discontinued the medication		3.2 (3)		4.8 (2)		1.9 (1)		< 0.001
Adhered to medication		55.5 (15)		72.2 (13)		22.2 (2)		< 0.001

^*^P-value was calculated by chi-square test & Fisher’s test

In the bi-variate analysis (see Table 3), ethnicity, parents’ education, income levels and employment status, were significantly associated with the parents’ decision to seek clinical diagnosis for their children with ADHD. Parents with higher levels of education, higher incomes and of Jewish ethnicity were more likely to seek diagnosis. Parental decisions whether to treat their ADHD positive children with medication and adherence to medication were also significantly associated with ethnicity, parents’ education and income levels.

**Table 3:** Variables associated with parents’ decision to seek clinical diagnosis, pharmacological treatment for their child with ADHD and adherence to medication

Variable		N = 94	P-value	N = 94	P-value	N = 94	P-value
		Seeking clinical diagnosis % (N)		Received pharmacological treatment % (N)		Adherence % (N)	
**Ethnicity**	Jewish	76.2 (32)	< 0.001	35.7 (15)	< 0.001	31 (13)	< 0.001
	Arab	36.5 (19)		5.8 (3)		3.8 (2)	
**Parents’ education**	Non-Academic	22.9 (8)	< 0.001	8.6 (3)	0.045	5.7 (2)	0.037
	Academic	72.9 (43)		25.4 (15)		22 (13)	
**Parents’ Income level**	Low – medium	35 (14)	0.001	5 (2)	0.003	5 (2)	0.013
	High	68.5 (37)		29.6 (16)		24.1 (13)	
**Employment**	Unemployed	30 (9)	0.001	10 (3)	0.123	6.7 (2)	0.09
	Employed	65.6 (42)		23.4 (15)		20.3 (13)	

Multivariable logistic regression was conducted to examine the association between the dependent variables (seeking clinical diagnosis, pharmacological treatment, and adherence to medication) and ethnicity, after adjusting for parents’ education, parents’ income levels and employment status, among children with ADHD. Two models are presented for each dependent variable. The first model presents the results before adjusting for parents’ education, and the second model after adjusting for parents’ education (see Table 4).

**Table 4:** Logistic Regression-Variables associated with parents’ decision to seek clinical diagnosis, parents’ decision to pharmacologically treat their children with ADHD and adherence to medication among ADHD positive children (N = 94)

		Seeking diagnosis OR (CI 95%)		Pharmacological treatment OR (CI 95%)		Adherence to medication OR (CI 95%)	
		Model 1 (Adjusting for ethnicity, income and employment)	Model 2 (Adjusting for ethnicity, education, income and employment)	Model 1 (Adjusting for ethnicity, income and employment)	Model 2 (Adjusting for ethnicity, education, income and employment)	Model 1 (Adjusting for ethnicity, income and employment)	Model 2 (Adjusting for ethnicity, education, income and employment)
**Ethnicity**	**Arab**	1	1	1	1	1	1
	**Jewish**	5.55 (2.24–13.76)*	2.082 (0.26–16.39)	9.07 (2.41–34.15)*	7.61 (1.14–50.86)*	11.2 (2.36–53.19)*	10.19 (1.18–88.01)*
**Education**	**Non-academic education**	-	1	-	1	-	1
	**Academic education**	-	6.14 (1.74–21.71)*	-	1.009 (0.06-17.00)	-	2.82 (0.06–17.88)

*p < 0.05. CI, confidence interval; OR, odds ratio

For the first dependent variable (parents’ decision to seek clinical diagnosis for their ADHD child), model 1 indicates that parents’ decision for seeking clinical diagnosis for their children was significantly associated with ethnicity, suggesting that Jewish parents were more likely to seek clinical diagnosis for their child with ADHD compared to Arab parents (P < 0.001, OR = 5.55, CI [2.24–13.76]. After adding education (model 2), the association between seeking clinical diagnosis and ethnicity was not significant, therefore we suggest that education can explain the difference between Arabs and Jews in seeking diagnosis and eliminates the association with ethnicity. The gaps between Arab and Jewish parents were explained by parents’ education (P = 0.005, OR = 6.14, CI [OR = 6.14, CI 1.74–21.71]. Parents’ decision to treat their children with medication for ADHD was associated only with ethnicity in both models, (P = 0.036, OR = 7.61, CI [1.14–50.86], as Arab children were 7.61 times more likely not to receive pharmacologic treatment. Similarly, adherence to medication was associated only to ethnicity (P = 0.035, OR = 10.19, CI [1.18–88.01].

## Discussion

The management of ADHD involves a series of steps that begins with awareness on the part of both parents and teachers, followed by diagnosis, and finally, the child’s willingness to adhere to medication. Parents play a central role in this process, as their perception of their child’s problematic behaviors influences their understanding of the need for treatment [26, 27]. Teachers also play a crucial role, as they are often the ones who advise parents to seek clinical help [28]. Ultimately, clinicians assess the condition and present all available treatment options [29].

In this study, the estimated prevalence of ADHD was similar among Jewish and Arab Muslim children. However, lower rates of parents seeking clinical diagnosis for their children with ADHD were reported by Arab parents compared to Jewish parents. Hence, a significantly lower percentage of Arab parents reported that their children were clinically diagnosed, and a higher percentage of Jewish parents reported treating their child with ADHD medication.

These results suggest that the difference between Arabs and Jews is not in the prevalence of ADHD but in diagnosis and treatment, as the estimated prevalence of ADHD was similar among Jewish and Arab Muslim children. This finding conflicts with previous studies in Israel that showed higher prevalence of ADHD among Jewish children [19–21]. In these previous studies, the prevalence of ADHD in school age children was estimated by rates of methylphenidate prescriptions. Calculating prevalence of ADHD by rates of methylphenidate prescriptions may be biased since not all parents approach a doctor to seek a clinical diagnosis and not all diagnosed children are treated with methylphenidate.

Various approaches for identifying children with ADHD have been presented in the literature. These include using medical records for diagnosis of ADHD or medical records for prescription medication use. Other studies have relied on parents reporting that their children have been formally diagnosed with ADHD, or surveys with parents using various diagnostic tools and using multiple reports from both parents and teachers [14, 30].

Observations of large studies from school-based samples in the United States, which relied on documentation in medical records of diagnosis or medication use, indicated lower rates of ADHD diagnoses among ethnic minorities [14, 31]. However, studies that estimated the prevalence of ADHD based on reports from teachers and parents found similar rates of ADHD prevalence among Caucasian and African American children [30]. Our study suggests similar results.

When examining the association between ethnicity and socioeconomic factors, with parents’ decision to seek health services for their child with ADHD, Arab parents were less likely to approach a doctor compared to their Jewish counterparts. Our findings suggest that parents with higher education levels among Arab and Jewish participants were more likely to seek clinical diagnosis for their child with ADHD. Studies have shown that there is a positive correlation between parents’ education and the utilization of mental health care services [32]. Generally, education is considered an important social determinant of health, as higher educational attainment is associated with higher incomes and wider access to healthcare. Parents with higher education levels may have better awareness and knowledge of their child’s condition, they are more likely to recognize when ADHD symptoms significantly interfere with academic, social and occupational functioning and may be more proactive in seeking appropriate treatment. Moreover, education enhances communication skills, as higher education levels enable patients to communicate more effectively with healthcare providers and teachers, leading to more comprehensive and detailed explanations of diagnosis and treatments [33–35]. Education equips individuals with necessary tools to navigate healthcare systems and make informed decisions about the health of their children [36]. In this regard, the Arab population in Israel is considered disadvantaged in almost every socioeconomic indicator, most importantly lower income, higher level of unemployment, and lower educational attainment [37]. Even though the sample of parents in the study had higher socioeconomic status in comparison to the total populations of both Arabs and Jews, among the study population, Arab participants had lower educational and income levels than Jewish participants. The difference in education levels between Arabs and Jews [38] may explain the observed differences in seeking diagnosis between the two ethnic groups.

The prevalence of pharmacological treatment in this sample was higher among Jewish children compared to Arab children. These results are similar to National studies of prescription medication use, that reported less use among African American and Hispanic children than white children [39]. In our study, ethnicity was the only variable associated with parents’ decisions to pharmacologically treat their children with ADHD. Other socioeconomic variables including parents’ education and parents’ income level were not associated medication in the logistic regression. This is consistent with a previous study that found lower rates of medication use among African American children which was attributed to race or ethnicity [31].

Ethnicity plays a critical role in the help-seeking process, affecting diagnosis, treatment and adherence. Ethnical/ racial differences may partially explain disparities in medical utilization between ethnic groups. Previous studies found that black parents are less likely to conceptualize ADHD as a medical condition requiring treatment. They perceive ADHD symptoms as behaviors that are better addressed by parenting and discipline, and therefore medical treatment is less acceptable among them [27, 40]. In addition, they are less likely to prefer medication treatment for ADHD than white parents, which appears to stem from misconceptions about the pharmacological treatment, greater concerns about side effects, less certainty about the efficacy of these medications and fear of social stigma [41, 42]. A recent qualitative study, demonstrated differences between Arab and Jewish mothers and their perceptions of ADHD. Arab mothers referred to ADHD in behavioral terms while Jewish mothers related to ADHD as a medical matter. Mothers’ acceptance of the ADHD diagnosis as a medical matter allowed them to understand their child’s problems and this helped them agree to administer medication to their child [43].

The current study suggests that adherence to medication is also associated with ethnicity, as adherence is much lower among the Arab minority. These results are in concordance with other studies which revealed that ethnic minority children are more likely to demonstrate low adherence and early discontinuation of ADHD medications [42]. Differences in prescription rates seem to be associated with negative cultural views of treatment outcomes or side effects [44, 45]. Furthermore, acceptance of the diagnosis and treatment may predict parents’ compliance with medical treatment [46].

Ethnicity and parental socioeconomic status (SES) have been shown to be related to help-seeking and may explain the between group differences in ADHD diagnoses, and its effects on health inequalities [47, 48]. In addition, cultural differences in parents’ perceived need for health care may partially explain disparities in utilization among various ethnic groups [49–51].

Parents’ decision to pharmacologically treat their child is a complex and multi-faceted process influenced by various individual, social and cultural factors. Minority groups report more barriers to seeking mental treatment. These barriers include self and social stigma, cultural beliefs against treatment, lack of cultural and linguistic adaptation of services, and cost and transportation problems [52, 53]. Cultural attitudes and beliefs surrounding ADHD may contribute to stigma and discrimination within specific populations. This stigma can discourage families from seeking help. The Arab world is characterized by enduring stigmas against mental illness and non-traditional mental health treatments [53]. This can be similar to members of Ultra-Orthodox Jewish community, as they generally under-utilize public mental health services, for fear of stigma [54]. In terms of cultural preferences, different cultures have unique beliefs and norms that shape the perception of ADHD symptoms. Symptoms may manifest differently within a cultural context [55].

It’s important to note that in Israel, medical services are available for all Israeli residents by the National Health Insurance (NHI) Law. All individuals in Israel are covered by a comprehensive health insurance including physician services, hospitalization, medication, and other services [56]. Every citizen is free to choose among four non-profit Health Maintenance Organizations (HMO). These HMOs provide their members with access to a package of health services that is specified within the NHI Law [57].

Although in Israel, Arabs have equal access to healthcare, in practice, a different pattern of use of these services is observed in the two communities. Jews utilize the specialist services more often than Arabs [58]. We assume that other factors could account for the Arab disparity. These factors include difficulty accessing mental health care services despite insurance, parental distrust of mental health professionals and parental attitudes and knowledge regarding ADHD. A recent study in Israel demonstrated that mental health services are less available in the Arab villages and cities, and public transportation services are inadequate in Arab areas which makes access to services in other towns difficult. Moreover, mental health services are not culturally adapted and include fewer Arab professionals, and Arab patients are less likely to seek treatment from Jewish therapists because of language, culture, and political barriers [52].

This study has several methodological limitations; first: the sample size of children with ADHD is relatively small. Second: selection bias, conducting the study required engaging consent from several parties including the school principals, homeroom teachers and the parents. Obtaining cooperation from the relevant parties and gaining their consent to participate in the study was more difficult among the Jewish population, resulting in lower response rates. Therefore, the Jewish sample is only partially representative of the general Jewish population, because it included mainly high socio-economic and highly educated parents, though we tried to engage participants from schools of lower socio-economic levels. Third, the study is based on parent and homeroom teacher reports, and was not confirmed by medical records or clinical assessment. This may introduce a recall bias. On the other hand, estimating ADHD prevalence by referring to medical reports would not enable us to reach children who have ADHD but are not diagnosed. So, estimating ADHD prevalence through elementary schools enabled us to reach children who are not diagnosed. Combining teacher and parent information may yield the strongest psychometric approach for identifying cases in a community [30]. In addition, previous studies have shown that parents’ and teachers’ reports of an ADHD diagnosis result in similar prevalence estimates to those attained through analysis of administrative claims data, suggesting convergent validity of estimated prevalence from both of these data sources [45]. Multiple reports are critical because DSM-5 emphasizes that impairment from ADHD symptoms must be present in at least two settings [2]. Clinical guidelines emphasize the importance of using teacher ratings when diagnosing ADHD [59]. Fourth, our cross-sectional methodology prevents us from inferring causal relationships, therefore a longitudinal study may help understand the factors affecting parents’ decision to seek clinical diagnosis and pharmacological treatment. Lastly, In the current study, we did not measure the access to mental health services by the studied families. Future studies should include a measure estimating barriers to mental health services among Arab minority in Israel.

## Conclusions

To conclude, diagnosis and treatment of ADHD is of high importance. This study exposes the gaps between the Arab and Jewish populations in seeking medical services in order to diagnose and treat ADHD, which will enable intervention programs to develop culturally suitable medical services, increase diagnosis and treatment of ADHD among children of minority groups and minimize the existing gaps. Overall, education plays a critical role in identifying children with ADHD. Low educated parents and minority groups should be targeted for interventions to increase awareness regarding ADHD and its treatment. Arab minorities are less likely to receive medical treatment for ADHD compared to their Jewish counterparts. Factors such as cultural beliefs and attitudes towards mental health, access to healthcare and language barriers may contribute to these gaps in utilization. It is important for healthcare providers to recognize and address these disparities to ensure that all children receive the necessary care for ADHD, regardless of their ethnicity.

Our results can be generalized to other minorities worldwide as inequality in diagnosing and treating minorities is not uniquely an Israeli phenomenon. A study focusing on attitudes and knowledge concerning ADHD diagnosis and treatment may contribute to the understanding of the gaps between different ethnic groups in Israel and throughout the world.

## Data Availability

The datasets generated and/or analyzed during the current study are not publicly available due to participants’ confidentially, but are available from the corresponding author on reasonable request.

## Abbreviations

ADHD: Attention Deficit Hyperactivity Disorder
DSM-5: Diagnostic and Statistical Manual of Mental Disorders, 5th Edition
ADHD-RS-V: ADHD– Rating Scale-V
